# Supplementary material for: Automated MRI system for clinically significant prostate cancer detection development validation and real-world implementation
Source: Nat Commun. 2025 Nov 23;16:11583. doi: 10.1038/s41467-025-66593-z (PMC12749140; doi:10.1038/s41467-025-66593-z)
Supplement: Supplementary file 1 — Supplementary Information [file 41467_2025_66593_MOESM1_ESM.pdf]

Supplementary material

Contents

Supplementary Note 1. Patient demographics, data sources, and case lists....................3

Supplementary Note 2. Segmentation of ProAI....................4

Supplementary Note 3. Pro-AI’s diagnostic performance with segmentation errors....................6

Supplementary Note 4. Inter-observer Agreement Assessment of PI-RADS....................7

Supplementary Note 5. Sample Size Estimation....................8

Supplementary Note 6. Clinical Information....................9

Supplementary Note 7. Histopathology-MRI Correlation Protocol....................10

Supplementary Note 8. Development of ProAI model....................11

Supplementary Note 9. Multi-reader multi-case (MRMC) study....................15

Supplementary Note 10. System implementation and processing time....................16

Supplementary Figure 1 The Workflow of development, validation, and real-World Implementation of AI System....................17

Supplementary Figure 2. Diagnostic Performance of ProAI and PI-RADS in External Test Sets (Patient Level, n=1,099)....................18

Supplementary Figure 3. Diagnostic Performance of ProAI and PI-RADS in Validation and External Test Sets (Lesion Level, n=2,107)....................19

Supplementary Figure 4. Representative cases of ProAI diagnostic performance with segmentation errors....................20

Supplementary Figure 5. Diagnostic Performance of PI-RADS and ProAI within Subgroups Stratified by Different Resolutions of T2WI and DWI in datasets test 1-5 and TCIA (Patient Level, n=879)....................21

Supplementary Figure 6. Analysis of the Consistency of PI-RADS Scores....................22

Supplementary Figure 7. Alluvial plot illustrating the shift in the distribution of PI-RADS scores before and after ProAI assistance in MRMC study (Patient Level, n=250)....................23

Supplementary Figure 8. Agreement with/without AI by reader level in MRMC study (Patient Level, n=250)....................24

Supplementary Figure 9. Paired sample t-test comparing nine doctors' diagnosis time with and without AI assistance in MRMC study (Patient Level, n=250)....................25

Supplementary Figure 10. Detailed architecture and workflow of the ProAI classification model....................26

Supplementary Figure 11. Illustrates the system architecture and integration pathways, along with screenshots of the user interface in different integration scenarios. 27

Supplementary Table 1. Comparison of Dice Values for Three Segmentation Algorithms 28

Supplementary Table 2. Detection Rates by PI-RADS≥3 and Lesion Characteristics in the test set 1-5 (Patient Level, n=515). 29

Supplementary Table 3. Segmentation Precision of Detected Lesions by PI-RADS≥3 in the Test Set 1-5 (Patient Level, n=485). 30

Supplementary Table 4. Distribution and Diagnostic Efficacy of Lesions with Different Dice Values in Test Set 1-5 (Patient Level, n=611). 31

Supplementary Table 5. Comparison of lesion-level diagnostic performance between the AI model and PI-RADS in test datasets (Lesion Level, n=2,107) 32

Supplementary Table 6. Subgroup Analysis of ProAI and PI-RADS Scores by T2WI/DWI Spatial Resolution, Grouping Criteria, and Machine Model in the Test Set 1-5 and TCIA (Patient Level, n=879). 33

Supplementary Table 7. T2WI Resolution-Based Performance Analysis in the Test Set 1-5 and TCIA (Patient Level, n=879). 34

Supplementary Table 8. DWI Resolution Based Performance Analysis in the Test Set 1-5 and TCIA (Patient Level, n=879). 35

Supplementary Table 9. Field Strength Performance Analysis in the Test Set 1-5 (Patient Level, n=839). 36

Supplementary Table 10. Diagnostic performance in the MRMC study (Patient Level, n=250). 37

Supplementary Table 11. Diagnostic Performance of PI-RADS in MRMC study (Patient Level, n=250). 38

Supplementary Table 12. The expertise of individual readers in the MRMC study (Patient Level, n=250). 39

Supplementary Table 13. Technology Acceptance Model (TAM) Questionnaire Results 40

Supplementary Table 14. Analysis of Diagnostic Inconsistencies Between ProAI and Radiologists in High-Risk Prospective Cohort (Patient Level, n=1,051). 41

Supplementary Table 15. Analysis of Diagnostic Inconsistencies Between ProAI and Radiologists in Low-Risk Prospective Cohort (Patient Level, n=1,051). 42

Supplementary Table 16. Scanning machines and scanning parameters of all datasets (Patient Level, n=7,849). 43

Supplementary Note 1. Patient demographics, data sources, and case lists

1.1 Patient Demographics

The training set was comprised of 2,794 cases from Hospital 1 and 1,500 cases from PI-CAI, with a mean age of 64.3 ± 10.7. This included 2,435 cases of nonPCa, 292 cases of ncsPCa, and 1,567 cases of csPCa. An independent validation set was also established, which included 189 cases of nonPCa, 22 cases of ncsPCa, and 267 cases of csPCa.

For the external tests, the patients came from five Chinese hospitals and an American public dataset of The Cancer Imaging Archive (TCIA): Test set 1 with 246 cases (mean age 70.0±9.2), including 104 nonPCa, 23 ncsPCa, and 119 csPCa cases; Test set 2 with 156 cases (mean age 68.9±8.6), comprising 73 nonPCa, 6 ncsPCa, and 77 csPCa cases; Test set 3 with 41 cases (mean age 68.8±8.5), including 25 nonPCa, 34 ncsPCa, and 12 csPCa cases; Test set 4 with 51 cases (mean age 68.1±8.2), consisting of 26 nonPCa, 6 ncsPCa, and 19 csPCa cases; and Test set 5 with 345 cases (mean age 66.9±8.4), including 190 nonPCa, 27 ncsPCa, and 128 csPCa cases; Test TCIA with 260 cases (mean age 65.7±7.5), comprising 67 nonPCa, 41 ncsPCa, and 152 csPCa cases.

Additionally, in the fourth phase of real-world clinical application, 927 consecutive cases (mean age 66.5 ± 8.1) were included between July and December 2023, prior to ProAI implementation. Following ProAI implementation between January and June 2024, 1,051 cases (mean age 66.2 ± 9.3) were included.

1.2 Data Source for the TCIA Test Set

This study utilized a single publicly available dataset for its external test analysis:

Collection Name: Prostate-MRI-US-Biopsy

Link: https://www.cancerimagingarchive.net/collection/prostate-mri-us-biopsy/

Initial Number of Cases: 1151 patients

Final Number of Cases: 260 patients

Exclusion criteria: (1) Duplicate patients or incomplete information; (2) multiparametric MRI performed more than 8 weeks prior to tissue sampling (3) prior androgen deprivation therapy, focal therapy, radiotherapy, or chemotherapy; (4) suboptimal MRI quality or incomplete sequences; and (5) non-prostatic malignancies (bladder cancer, sarcomas) with prostatic involvement.

The exact list of patients included in the final analysis is provided in the FigureShare: Source Data_TCIA_case_list.csv.

Supplementary Note 2. Segmentation of ProAI

2.1 ProAI Achieves High Dice Similarity Coefficients for Prostate Segmentation and PI-RADS-Dependent Lesion Segmentation Performance Across all sets

During the segmentation task, the DSC for prostate segmentation achieved an accuracy of 0.956 (IQR: 0.949, 0.964) in the training set, 0.931 (IQR: 0.926, 0.939) in the validation set and 0.925 (IQR: 0.836, 0.951) in the test set. Furthermore, the ProAI demonstrated a median DSC of 0.734 (IQR: 0.401, 0.867) for lesion segmentation in the training set, 0.764 (IQR: 0.543, 0.889) in the validation set, and 0.564 (IQR: 0.388, 0.730) in the test set. In addition, the higher the PI-RADS score of the dominant lesion, the higher the segmentation DSC of the lesion.

2.2 Analysis of Lesion Detection and Segmentation Precision

We conducted an in-depth analysis of the lesion detection capabilities and segmentation precision of our model beyond the standard Dice similarity coefficient (DSC) metrics. Using a threshold of Dice value > 0.1 to consider a lesion as "detected" (regardless of boundary precision), we analyzed detection rates across PI-RADS categories in the external test sets:

(1) PI-RADS 3 lesions: 87.9% detected (152/173)

(2) PI-RADS 4 lesions: 96.4% detected (133/138)

(3) PI-RADS 5 lesions: 98.0% detected (200/204)

(4) Overall detection rate (PI-RADS ⩾3): 94.2% (485/515)

For missed lesions (those with DSC = 0), qualitative analysis revealed common characteristics:

76.7% (23/30) of missed lesions were PI-RADS 3

53.3% (16/30) were small (<7mm in maximum diameter)

70.0% (21/30) were located in the base or apex of the prostate

46.7% (14/30) had diffuse or indistinct margins on mpMRI

For detected lesions, we categorized boundary precision as follows:

High precision (DSC > 0.7): 41.6% (202/485)

Moderate precision (DSC 0.4-0.7): 35.3% (171/485)

Low precision (DSC 0.1-0.4): 23.1% (112/485)

To assess potential utility for targeted biopsy guidance, we calculated the centroid distance between predicted and ground truth lesions:

Median centroid distance for detected lesions: 2.8mm (IQR: 1.5-4.3mm)

Proportion of lesions with centroid distance <5mm: 83.7% (406/485)

False positive detections (regions segmented without corresponding ground truth) were characterized by:

False positive rate: 0.31 per patient

Mean size: significantly smaller than true lesions (0.18 vs. 0.54 cm^3, $p<0.001$)

Common locations: predominantly in transition zone (68%), often in areas of benign prostatic hyperplasia

These findings show that while the model achieves high detection rates for clinically significant lesions, segmentation precision varies considerably. However, the high proportion of lesions with accurate centroid localization suggests potential utility for targeted biopsy applications, even in cases with suboptimal boundary delineation.

Supplementary Note 3. Pro-AI’s diagnostic performance with segmentation errors

We conducted a detailed error analysis specifically focused on cases where the segmentation module performed sub-optimally. We identified 167 cases (20% of the total test set) with significant segmentation errors, defined as DSC < 0.4 compared to radiologist annotations. For these cases, the classification module maintained an AUC of 0.921 (0.874-0.968), only 37% (37/167) were high-risk cases. This indicates that the majority of segmentation errors occurred in benign patients, and ProAI still demonstrated high diagnostic efficacy. Details showed in Supplementary Table 4. Two cases of high-risk patients with csPCa but with erroneous AI segmentation are shown in Supplementary Figure 4.

Supplementary Note 4. Inter-observer Agreement Assessment of PI-RADS

The Cohen's kappa coefficients between the two radiologists were 0.577 (95%CI: 0.561, 0.593) in the training set, 0.652 (95%CI: 0.608, 0.695) in the validation set, 0.622 (95%CI: 0.590, 0.655) in the test set.

Supplementary Note 5. Sample Size Estimation

In the first phases, ProAI was expected to achieve an AUC of 0.85 in the test set. Meanwhile, senior radiologists, following the PI-RADS v2.1 guidelines, had an average AUC of 0.80 for diagnosing csPCa[1]. Set the parameters as follows in the “Tests for One ROC Curve” module: alpha = 0.05, the final statistical power was 0.8, the ratio (negative: positive) was 0.5, AUC0 = 0.80, and AUC1 = 0.85, and other default values. The required sample size for inclusion is 539 cases.

In the fourth phases, the AI model was expected to achieve an AUC of 0.85 on the prospective set. Meanwhile, senior radiologists, following the PI-RADS v2.1 guidelines, had an average AUC of 0.80 for diagnosing csPCa[1]. In the cohort of patients who received MRI scans at Hospital 1, the incidence of csPCa detection was 25%, predominantly among individuals with a prior elevation of PSA levels. Set the parameters as follows in the “Tests for One ROC Curve” module: alpha = 0.05, the final statistical power was 0.8, the ratio (negative: positive) was 3, AUC0 = 0.80, and AUC1 = 0.85, and other default values. Accounting for an estimated 10% loss to follow-up, the final sample size to be included was 1039.

In the multi-reader multi-case study (phase 2), the sample size calculation method used the RJafroc package in R (v4.6.1, R Foundation for Statistical Computing, USA). Jackknife was used to calculate the covariance, and the OR method was used to calculate the diagnostic efficacy of different readers with different diagnostic methods.

We carried out a pilot study with three radiologists and 100 cases to assess the correlation of diagnostic results both within and across readers using various methods. The expected difference in AUC values was 0.05 with nine readers (a significance level of 0.05, and a desired power of 0.9). Incorporating 20% redundancy to account for potential data loss, the final sample size to be included was 250. To ensure that the clinicians participating in this study are representative, we recruited three subgroups of clinicians including two senior radiologists, five general radiologists, and two urologists.

The design of the acceptance study (phase 3) does not involve comparing outcome variables, so estimating the sample size is unnecessary.

Supplementary Note 6. Clinical Information

The clinical information was derived entirely from the medical record system. The clinical data included: age, total serum prostate-specific antigen (t-PSA) within 8 weeks prior to biopsy, free serum prostate-specific antigen (f-PSA) within 8 weeks prior to biopsy, and the percentage of free prostate-specific antigen (f/t PSA).

MRI Scan and Analysis

Patients across all datasets underwent T2-weighted (T2WI), dynamic contrast-enhanced (DCE), and diffusion-weighted imaging (DWI).

The training set comprised patients from Hospital 1 and the public dataset (PI: CAI), while the internal validation set included only patients from Hospital 1. Five external test sets were sourced from five independent hospitals in China. MRI scans at Hospital 1 were conducted using 3 Tesla MR750, SIGNA Premier, and Skyra scanners without an endorectal coil. Detailed scanning equipment and parameters for all six hospitals and the public dataset are provided in Supplementary Table 5.

Supplementary Note 7. Histopathology-MRI Correlation Protocol

All MRI-pathology correlations were performed through a standardized process by a multidisciplinary team including a genitourinary radiologist (Y.B. with 15 years of experience) and a urological pathologist (H.J. with 12 years of experience). The correlation procedure varied according to the type of pathological specimen:

1. **For Radical Prostatectomy Specimens**: Whole-mount prostatectomy sections were sliced at 4mm intervals in the axial plane, perpendicular to the prostatic urethra, to match the orientation and slice thickness of axial T2-weighted MR images. The radiologist and pathologist jointly reviewed the MRI and pathological sections side by side to establish direct lesion correspondence.
2. **For Combined Systematic and Targeted Biopsies**: MR-ultrasound fusion platforms (UroNav, Philips/Invivo) provided precise spatial coordinates of each biopsy core, which were recorded and used for direct correlation with MRI findings. Post-biopsy documentation included a standardized mapping diagram recording the exact location of each core.
3. **For Systematic Biopsy Only**: We employed the standardized 12-core systematic biopsy template (6 cores from each lobe, covering apex, mid-gland, and base regions in both peripheral and transition zones). For cases where lesions were not clearly visible on imaging but cancer was detected on systematic biopsy, lesion location was determined based on the specific positive cores within the systematic biopsy mapping.

In cases where lesion location correlation was challenging (approximately 8.4% of cases), the following protocol was implemented:

1. **Multiple Small Foci on Pathology**: When multiple microscopic cancer foci were identified without a clear dominant lesion corresponding to the MRI finding, the case was reviewed in a multidisciplinary conference. If consensus could not be reached on exact correlation, the highest Gleason score from the anatomical region corresponding to the MRI finding was used.
2. **Discrepant Locations**: When the location of cancer on pathology appeared to differ from the suspicious region on MRI, we implemented a 'nearest-neighbor' approach, where correlation was established with the closest pathological focus within 10mm of the MRI-identified lesion.
3. **Quality Control**: 15% of all correlations were randomly selected for independent review by a second radiologist-pathologist pair to ensure consistency in the correlation process. The inter-observer agreement for lesion correlation was substantial (Cohen's kappa = 0.82)."

Supplementary Note 8. Development of ProAI model

8.1 Imaging Pre-Processing

Two radiologists with 8 and 9 years of diagnostic experience outlined the contour and lesions of the prostate gland in MRI for all patients in the training set. In cases of disagreement, a genitourinary radiologist (with 20 years of diagnostic experience) made the final decision. The lesions were outlined on T2WI, DWI, and ADC maps. During the delineation process, multiple sequences were referenced against each other and combined with radical surgical pathology or biopsy results.

The lesion delineation was performed on the training set, internal validation set and external validation set (Test 1-5). During the delineation process, high-risk lesions were annotated according to histopathology-MRI correlation protocol (Supplementary Note 7). Additionally, we delineated lesions (PI-RADS $\geqslant$2) in benign cases.

The algorithm encompasses several preprocessing steps: registration, cropping, and normalization.

Initially, using the world coordinates embedded in the DICOM files, the ADC and DWI slices were aligned with their corresponding T2WI slices. Subsequently, to streamline the field of view and enhance the computational efficiency of deep learning, the prostate region was centrally cropped for both prostate segmentation and prostate lesion segmentation tasks. For the lesion classification modules, the lesions themselves were centrally cropped. Lastly, normalization was applied: for each T2WI and DWI, all voxel values were standardized by subtracting the mean and dividing by the standard deviation. In the case of ADC, an additional step was taken where voxel values were divided by 1000, ensuring the preservation of the clinical significance of the numerical values.

Protocol Harmonization To address the heterogeneity of MRI acquisition protocols across centers, we implemented a comprehensive harmonization pipeline. Each module (segmentation, protocol harmonization, risk estimation) reads from the original images and applies its own optimal voxel size for algorithmic and computational reasons, not sequential re-resampling, thereby avoiding compounding interpolation errors. All images underwent preprocessing including N4 bias field correction, Z-score intensity normalization, and spatial resampling to consistent dimensions [3.0, 0.39, 0.39 mm]. The N4ITK algorithm was applied to correct for intensity non-uniformities in all MRI sequences, with the following parameters: number of iterations = 50, convergence threshold = 0.001, spline distance = 200mm. Each sequence underwent Z-score normalization separately, calculated as $z = (I - \mu)/\sigma$, where I represents the voxel intensity, μ is the mean intensity of the prostate region, and σ is the standard deviation. This normalization was performed independently for each sequence and each patient. Additionally, our training strategy explicitly incorporated protocol diversity through

augmentation techniques that simulate different acquisition parameters, enabling the model to learn protocol-invariant features. These augmentations included:

- Random intensity scaling (±15%) - Random gaussian noise addition (σ = 0-0.03)
- Random gamma correction (γ = 0.8-1.2)
- Random contrast adjustment (±10%)

Our network architecture includes parallel feature extraction paths for T2WI and DWI, with adaptive weighting mechanisms that can compensate when one sequence has suboptimal quality. We incorporated a feature normalization layer after initial convolutional blocks that adaptively normalized feature distributions from different scanners, helping to minimize scanner-specific characteristics while preserving pathology-related features.

8.2 Prostate and Lesion Segmentation

To enhance compatibility with data sourced from various institutions, our training dataset encompassed both T2WI and T2 fat-suppressed weighted imaging (FS-T2WI). Before training, all image data were resampled to achieve a uniform spacing of [4.0, 0.78125, 0.78125]. For prostate region segmentation, we adopted the U-Net network[2], a deep learning model originally introduced to address the challenges of biomedical image segmentation. The U-Net architecture features a symmetric U-shape, comprising a left-side encoding network and a right-side decoding network. The encoding network was responsible for extracting image features and context information, while the decoding network restored image resolution and localization details. These two networks were connected by skip connections that facilitate the fusion of features. The loss function adopts Dice loss and cross-entropy loss, defined as follows:

$$Loss = W_1 L_{dice} + W_2 l_c$$

Where is dice loss and is cross-entropy loss. and are weight coefficients. The model was implemented using nnU-Net[3], an automated and flexible deep learning framework for medical image segmentation. While our model does not use lesion size as an explicit input parameter (unlike PI-RADS which incorporates size thresholds), the 3D convolutional neural network architecture inherently captures spatial dimensions and volumetric information through feature extraction from the complete lesion volume.

(1) Prostate zonal segmentation: Prostate zonal segmentation was carried out using T2WI, identifying five classes: the Peripheral Zone, the Central Zone, the Transition Zone, the Anterior Fibromuscular Stroma, and the Background. This segmentation model, like the prostate segmentation, was trained using nnU-Net.

(2) Lesion segmentation: Building upon the U-Net model, T2WI, ADC, and multi b-value DWI were used as inputs to delineate lesions, encompassing proliferations and tumors. Like prostate segmentation, the training of this model was also conducted using nnU-Net. The model incorporated

three input channels: the first channel could be either T2WI or FS-T2WI, with the selection randomized during the training process; the second channel was ADC, which was subject to random dropping with a predefined probability during training; the third channel was DWI, where a b-value was randomly chosen during training. During the inference phase, the model exhibited flexibility in accepting various channel inputs, accommodating both T2WI and FS-T2WI sequences, managing missing channels, and adapting to diverse b-value sequences.

8.3 Low-Risk and High-Risk Classification

Model Architecture:

The classification model adopts HarDNet-39DS (Harmonic Densely Connected Network variant), a computationally efficient CNN architecture with three key characteristics: (1) Optimized dense connections reducing memory access costs by 40% compared to standard DenseNet; (2) Hybrid depth-wise and point-wise convolutions achieving 1.8× faster inference speed; (3) Preserved multi-scale feature fusion capability through harmonic connection blocks.

Input Data Processing:

The input includes multi-parametric MRI sequences:

T2-weighted imaging (T2WI), Apparent Diffusion Coefficient (ADC) map, multi-b-value Diffusion-Weighted Imaging (DWI) (b=1000/1500/2000/3000 s/mm²), and the lesion mask from segmentation. All sequences were standardized to uniform spatial resolution [3.0, 0.39, 0.39 mm] through linear interpolation. A 3D region of interest (ROI) with dimensions (16, 128, 128) voxels was cropped centering on each lesion, encompassing both the lesion core and perilesional tissue (5mm margin).

Training Strategy:

Protocol-Aware Augmentation:

Implemented random channel dropout (50% probability per DWI b-value) and T2WI/FS-T2WI alternation during training to enhance protocol robustness. The augmentation pipeline included:

Random intensity scaling (±15%)

Gaussian noise injection (σ=0-0.03)

Gamma correction (γ=0.8-1.2)

Multi-sequence misalignment simulation (±2mm)

Feature Normalization:

A scanner-adaptive normalization layer was inserted after initial convolutions, dynamically adjusting feature statistics using:

$$\mu\_f = \alpha\mu\_batch + (1\text{-}\alpha)\ \mu\_scanner$$

$$\sigma\_f = \alpha\sigma\_batch + (1\text{-}\alpha)\ \sigma\_scanner$$

where α=0.7 (learnable parameter) balances batch and scanner-specific statistics.

Multi-Source Training Data:

The training set combined:

Expert-annotated lesions (n=1,240): Manually segmented by radiologists

Model-annotated lesions (n=2,150): Auto-segmented benign lesions from negative cases

This dual-source approach improved model robustness to segmentation variability while maintaining diagnostic specificity (AUC increase from 0.82→0.87 in validation).

Case-level Malignancy Probability:

For clinical interpretability, the model outputs were transformed through temperature scaling calibration (T=0.85). Final case probability P_case was computed as:

$$P\_case = \max(P\_lesion1, P\_lesion2, ..., P\_lesionN)$$

where P_lesion represents calibrated probabilities for each lesion. This approach achieved 92% concordance with expert consensus in multi-lesion cases.

Supplementary Note 9. Multi-reader multi-case (MRMC) study

The 9 readers were randomly assigned to two groups (4:5). Among them, there were 2 senior radiologists (reader 1 and 2), 5 general radiologists (reader 4-5 are resident radiologists and 7-9 are associate radiologists), and 2 urologists (reader 3 and 6). Firstly, in June 2023, each reader was trained to understand the reading process and received professional training on PI-RADS v2.1. Then, the MRMC study design followed a randomized crossover pattern.

From July to September 2023, readings were done on specialized workstations with de-identified, prostate mpMRI (T2WI, ADC, high b-value DWI and DCE). At this workstation, ProAI's diagnostic results are delivered directly to physicians, who need only click to review them. 4 readers reviewed these cases with ProAI assistance and 5 readers without ProAI assistance from the beginning. To reduce bias from memory and reading order, the two sessions were separated by a 6-week washout period, during which readers continued their clinical duties. In the second session, cases were presented in random order, and readers had no access to clinical information, other readers' results, or pathological findings. Furthermore, the system automatically logged the duration each reader took to render a diagnosis in both phases of the diagnostic process, thereby enabling an efficient assessment of the radiologists' performance. All readers completed the assessments within a week, and the time taken for each case was also recorded.

The average Cohen's kappa coefficients between readers were 0.287 (95%CI: 0.276, 0.298) without AI, and it showed significant improvement with AI assistance (0.415 (95%CI: 0.403, 0.426), $p < 0.01$).

Supplementary Note 10. System implementation and processing time

The ProAI system was developed and trained on a system running Ubuntu 22.04 (Canonical Ltd., London, UK), equipped with 128 GB of RAM, a 12-core Intel Core i7-6800K CPU running at 3.4 GHz, and four NVIDIA GeForce GTX 1080 Ti GPUs, each with 11 GB of VRAM. The deep learning models were implemented using the PyTorch deep learning framework (PyTorch 2.0.0, http://pytorch.org). Medical imaging data annotation was performed with ITK-SNAP (version 3.8.0, http://www.itksnap.org), a widely used open-source tool for segmentation and visualization.

For clinical deployment, the system was implemented on a dedicated high-performance computing server with NVIDIA A100 GPUs (80GB), dual Intel Xeon processors (64 cores), and 256GB RAM. With this enhanced configuration, the average processing time from data upload to completion of ProAI diagnosis was approximately 90 seconds per case. This processing time includes data transfer, image preprocessing, prostate and lesion segmentation, and risk classification. The system supports batch processing, allowing multiple cases to be analyzed simultaneously.

In the clinical workflow, ProAI processing typically begins immediately after MRI acquisition is completed, occurring in parallel with other clinical processes. This ensures that AI results are ready for review when radiologists begin their interpretation session, thus not adding additional waiting time to the overall workflow. Notably, the AI processing times can vary depending on server load and configuration, and further optimization of the inference pipeline could potentially reduce processing times in future implementations. All tools and frameworks used in this study are open-source, promoting reproducibility, transparency, and accessibility in medical research.

References

1. Di Franco F*, et al.* Characterization of high-grade prostate cancer at multiparametric MRI: assessment of PI-RADS version 2.1 and version 2 descriptors across 21 readers with varying experience (MULTI study). *Insights Imaging* **14**, 49 (2023).

2. Osman YBM, Li C, Huang W, Wang S. Sparse annotation learning for dense volumetric MR image segmentation with uncertainty estimation. *Phys Med Biol* **69**, (2023).

Supplementary Figure 1 The Workflow of development, validation, and real-World Implementation of AI System.

Workflow diagram

AI= artificial intelligence; ProAI = the clinically significant prostate cancer artificial intelligence model; PI-CAI = prostate imaging: cancer artificial intelligence

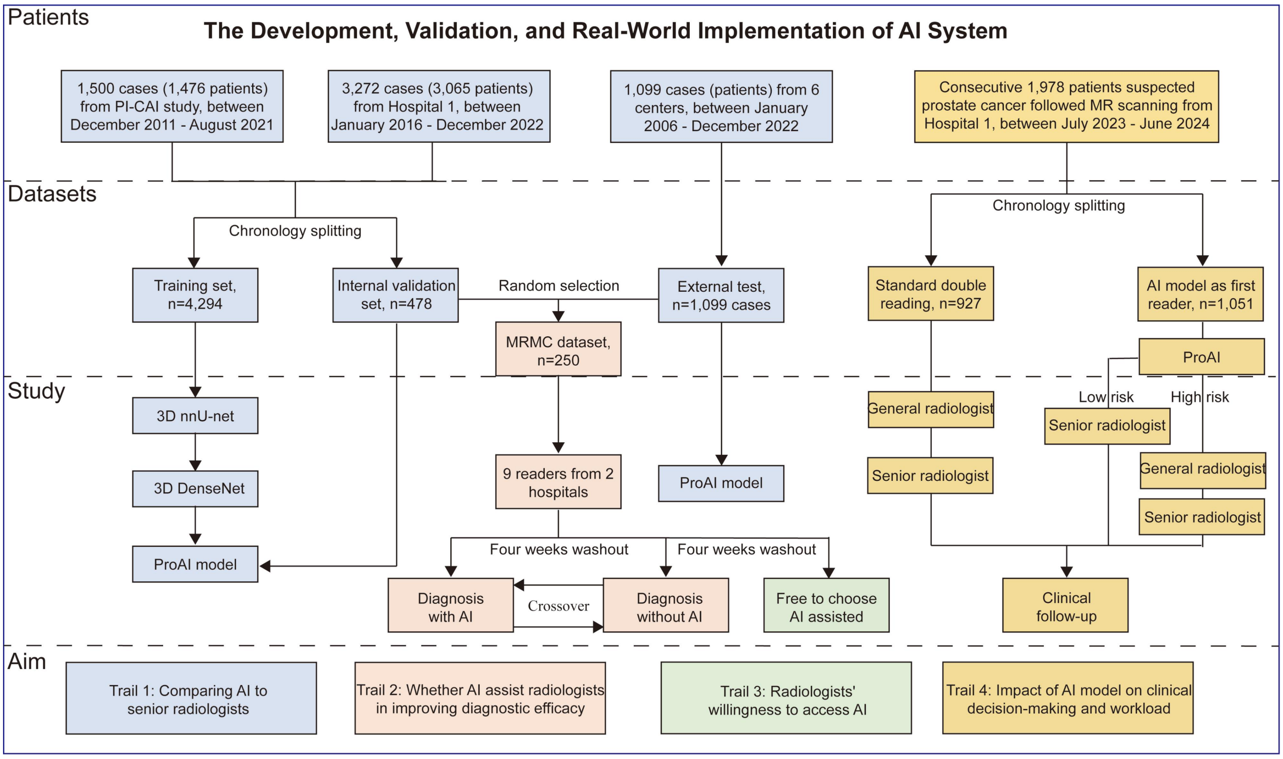

Supplementary Figure 2. Diagnostic Performance of ProAI and PI-RADS in External Test Sets (Patient Level, n=1,099)

(A-F) ProAI = the clinically significant prostate cancer artificial intelligence model; AUC = Area Under the Curve; PI-RADS = prostate imaging reporting and data system; Performance metrics: AUC was derived from continuous scores. Sensitivity/specificity utilized a fixed PI-RADS threshold (≥3); the ProAI threshold was dynamically optimized per dataset by maximizing Youden's J statistic. AUCs were compared using two-sided DeLong tests; exact p-values are provided in the Source Data.

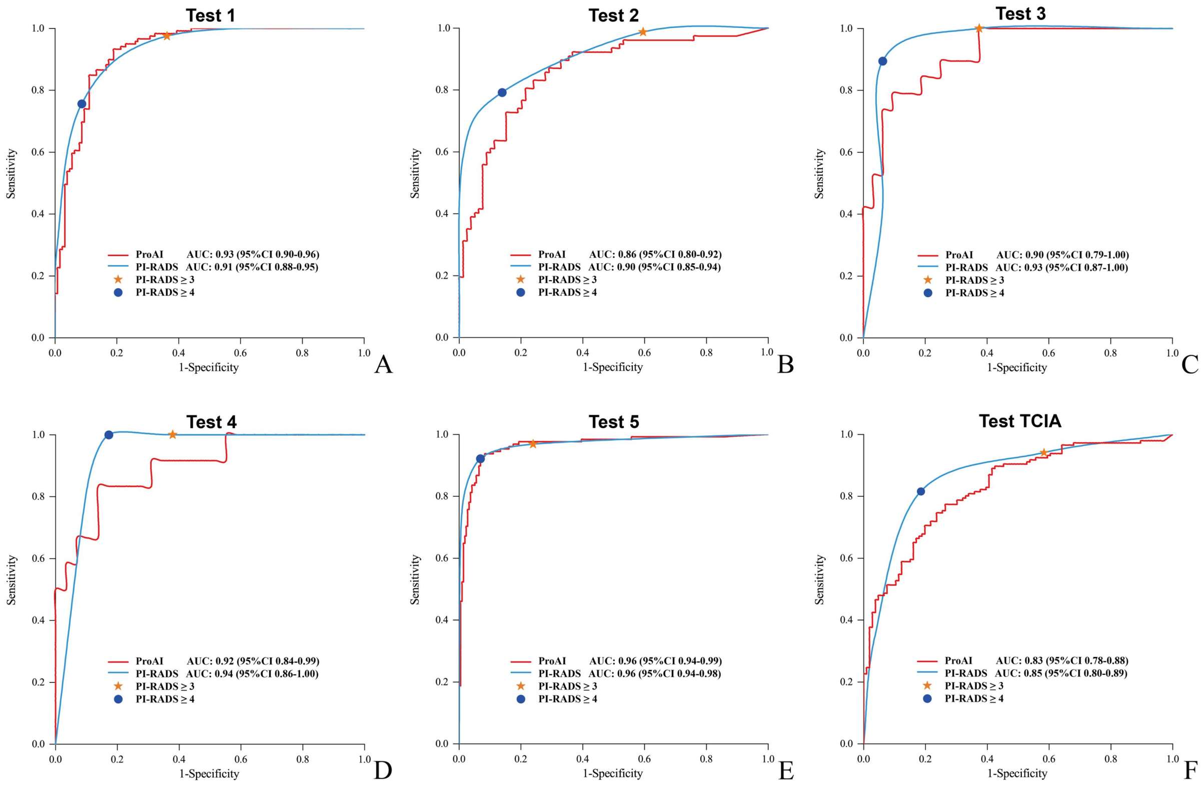

Supplementary Figure 3. Diagnostic Performance of ProAI and PI-RADS in Validation and External Test Sets (Lesion Level, n=2,107)

(A-H). ProAI = the clinically significant prostate cancer artificial intelligence model; AUC = Area Under the Curve; PI-RADS = prostate imaging reporting and data system; Performance metrics: AUC was derived from continuous scores. Sensitivity/specificity utilized a fixed PI-RADS threshold (⩾3); the ProAI threshold was dynamically optimized per dataset by maximizing Youden's J statistic. AUCs were compared using two-sided DeLong tests; exact p-values are provided in the Source Data.

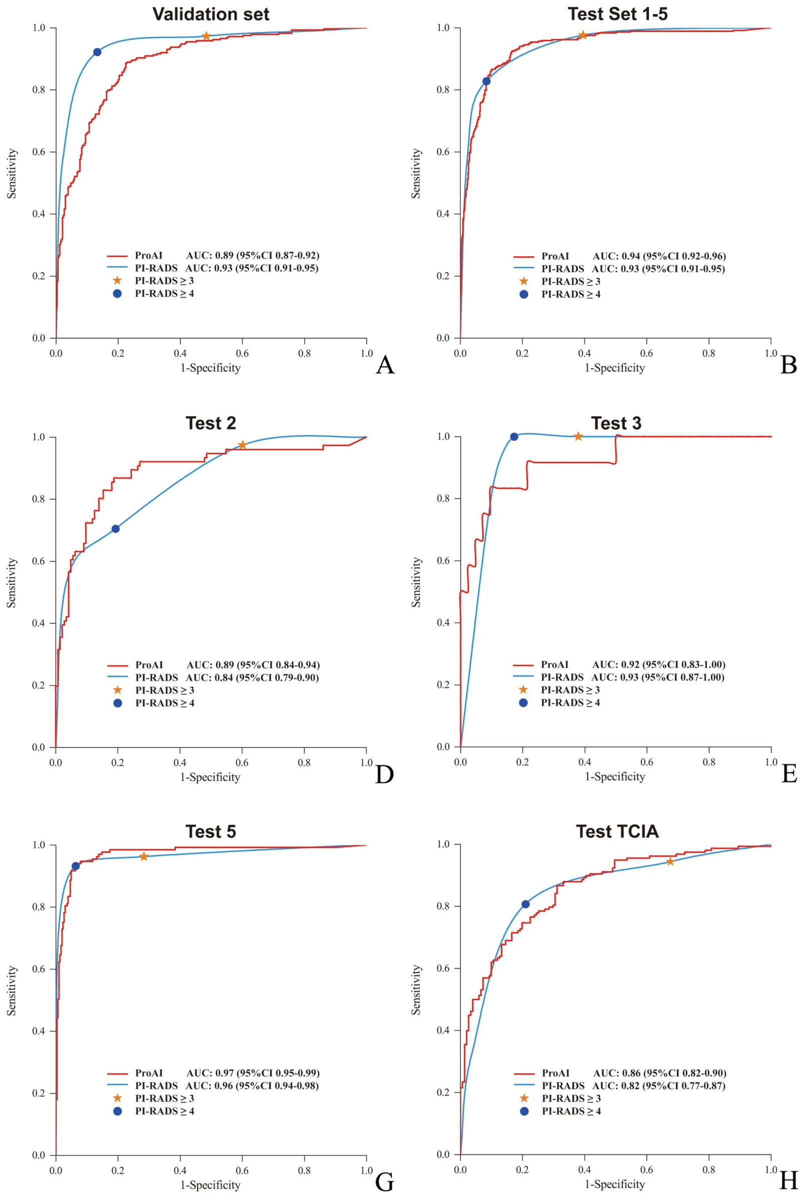

Supplementary Figure 4. Representative cases of ProAI diagnostic performance with segmentation errors

Case 1: (A-C) T2-weighted, high b-value DWI, and ADC map images. (D) Radiologist annotated lesion contour. (E) AI-generated lesion contour, and showed a low probability (0.44) for csPCa. AI failed to correctly segment the high-risk lesion, but still diagnosed it as benign at the lesion level.

Case 2: (F-H) T2-weighted, high b-value DWI, and ADC map images. (I) Radiologist annotated lesion contour. (J) AI-generated lesion contour, and showed a high probability (0.96) for csPCa. Although AI erroneously flagged extra-prostate artifacts as lesions, it correctly detected the actual lesion, leading to a correct final diagnosis. Representative clinical cases shown for illustration; repeat counts are not applicable.

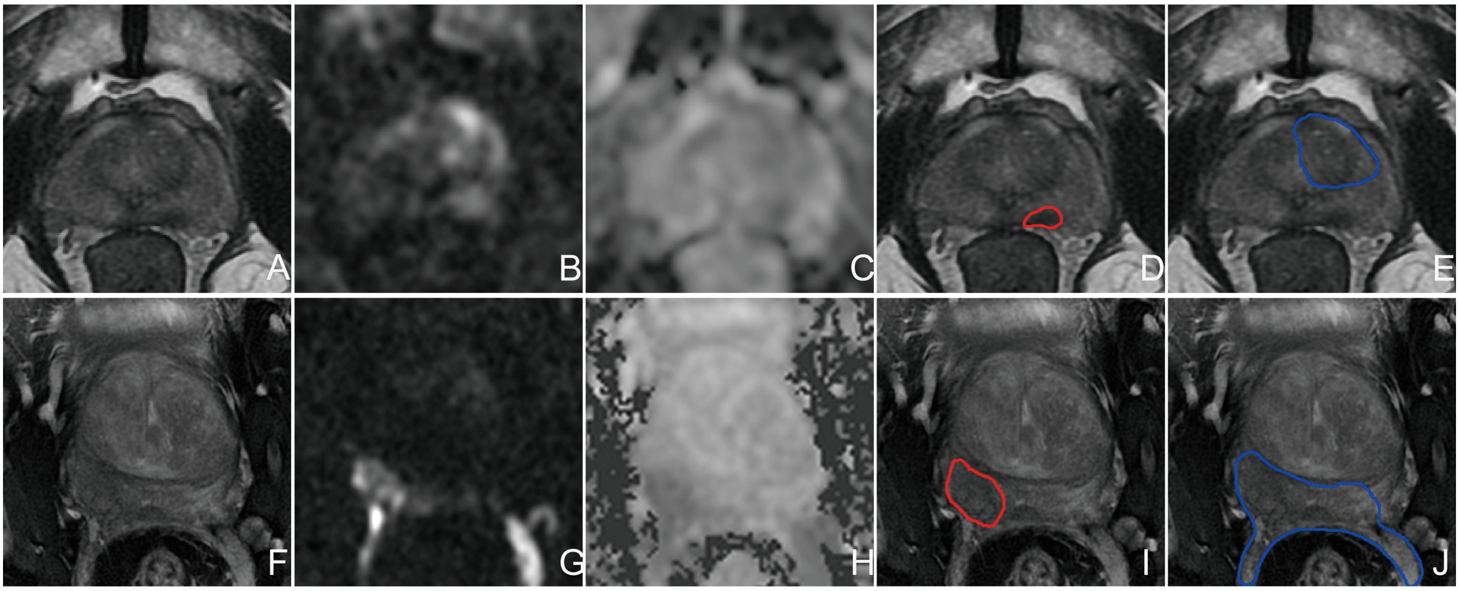

Supplementary Figure 5. Diagnostic Performance of PI-RADS and ProAI within Subgroups Stratified by Different Resolutions of T2WI and DWI in datasets test 1-5 and TCIA (Patient Level, n=879).

(A) ProAI at different T2WI resolutions; (B) PI-RADS at different T2WI resolutions; (C) ProAI at different DWI resolutions; (D) PI-RADS at different DWI resolutions. Performance metrics: AUC was derived from continuous scores. Sensitivity/specificity utilized a fixed PI-RADS threshold (≥3); the ProAI threshold was dynamically optimized per dataset by maximizing Youden's J statistic. AUCs were compared using two-sided DeLong tests; exact p-values are provided in the Source Data.

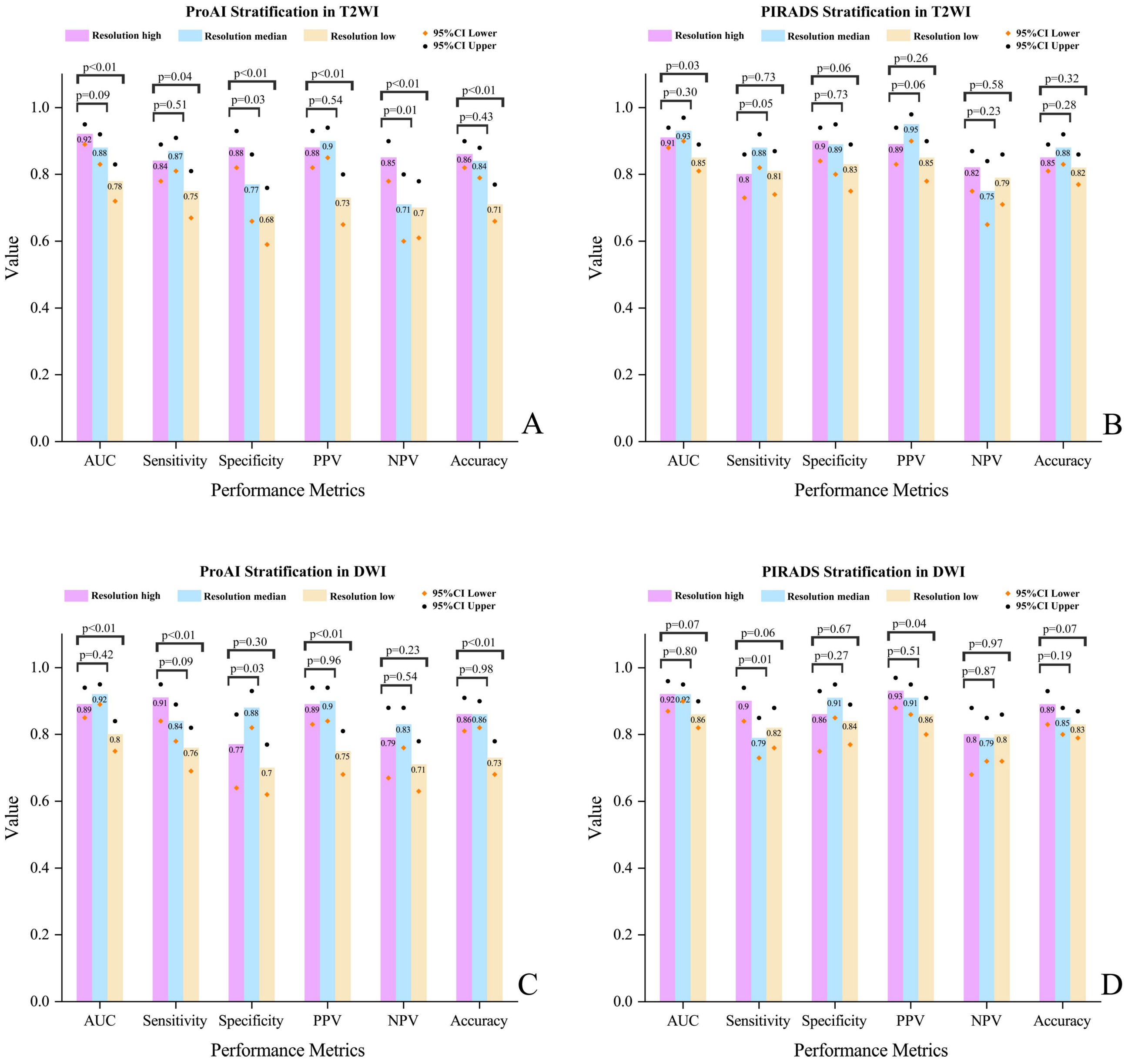

Supplementary Figure 6. Analysis of the Consistency of PI-RADS Scores.

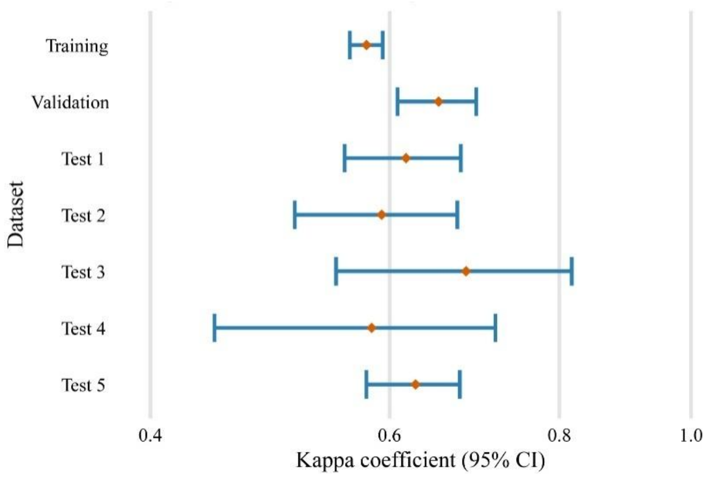

Supplementary Figure 7. Alluvial plot illustrating the shift in the distribution of PI-RADS scores before and after ProAI assistance in MRMC study (Patient Level, n=250).

(A - I) The diagnostic decision-making process altered for nine readers, with each experiencing a change facilitated by artificial intelligence assistance.

Low-risk = negative pathology or non-clinically significant prostate cancer; high-risk= clinically significant prostate cancer; Readers' assessments, ranging from scores 1 to 5, adhere to the guidelines in the PI-RADS v2.1 protocol.

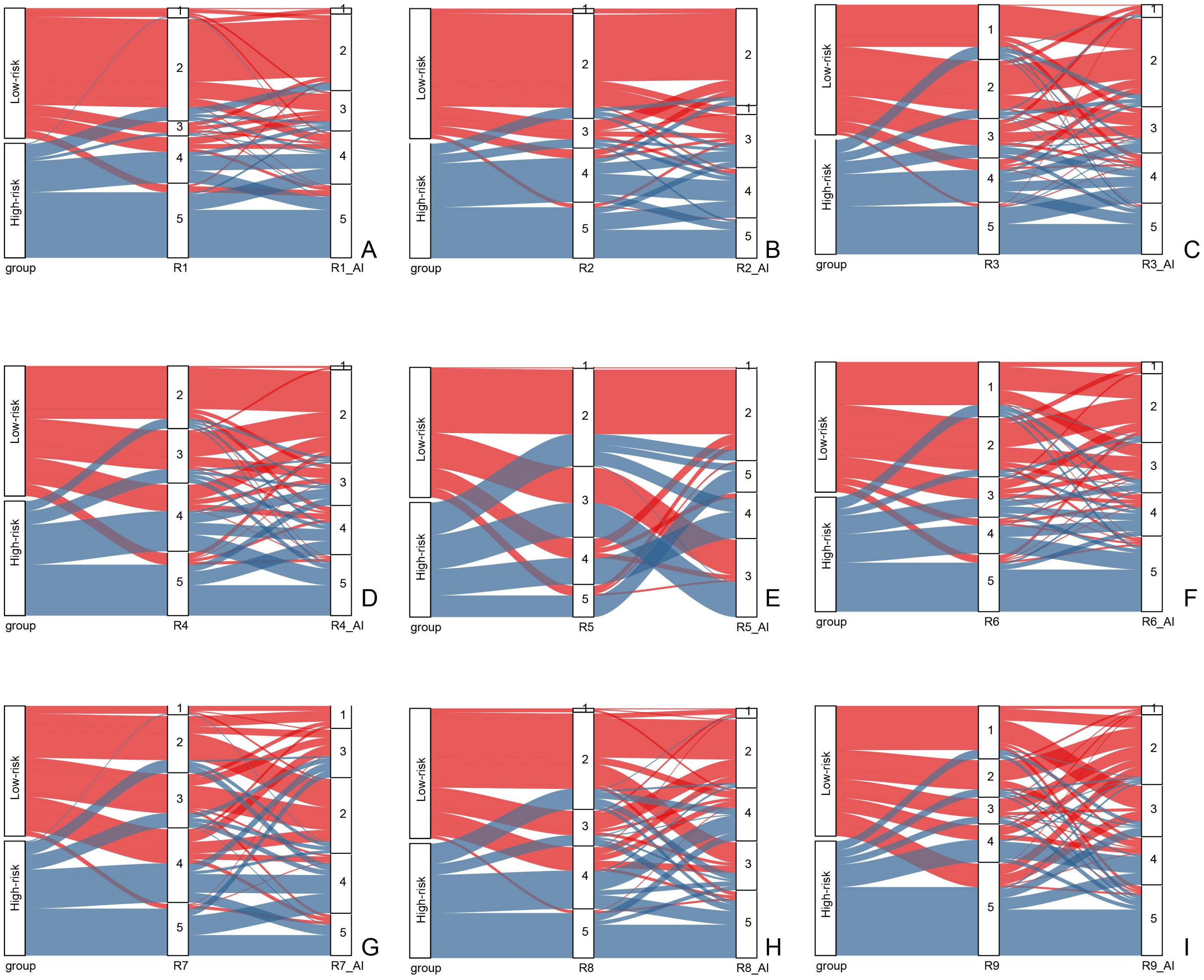

Supplementary Figure 8. Agreement with/without AI by reader level in MRMC study (Patient Level, n=250).

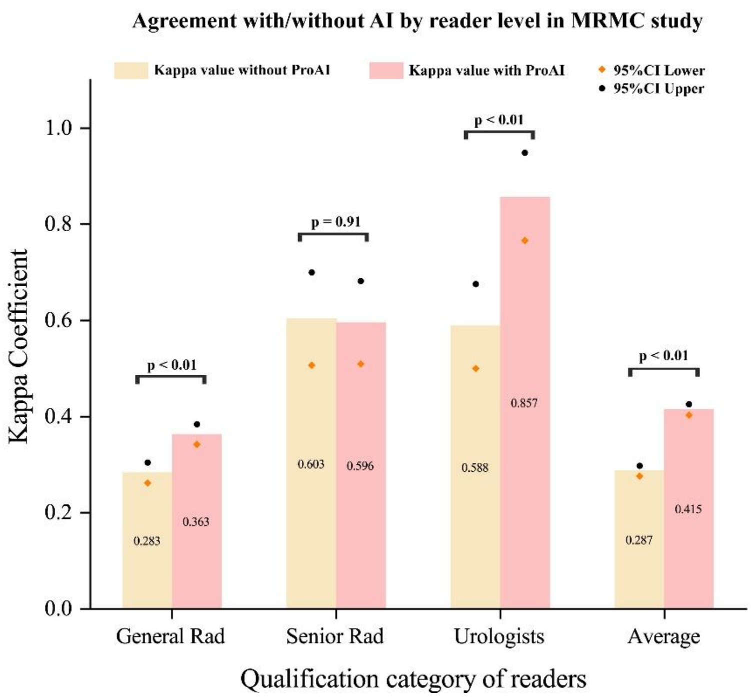

Supplementary Figure 9. Paired sample t-test comparing nine doctors' diagnosis time with and without AI assistance in MRMC study (Patient Level, n=250).

Black and red boxes indicate diagnostic times for independent reading and AI-assisted diagnosis, respectively. Changes in each case are shown by lines connecting the points. (A-I) represented the readers ranked from 1st to 9th. The diagnostic times for all readers, with the exception of reader 1, significantly decreased following AI assistance. Time per case is reported in seconds; lines connect paired readings per case; symbols are defined in the legend. Within-reader differences were assessed by two-sided paired t-tests; exact p-values are provided on the figure or in the Source Data.

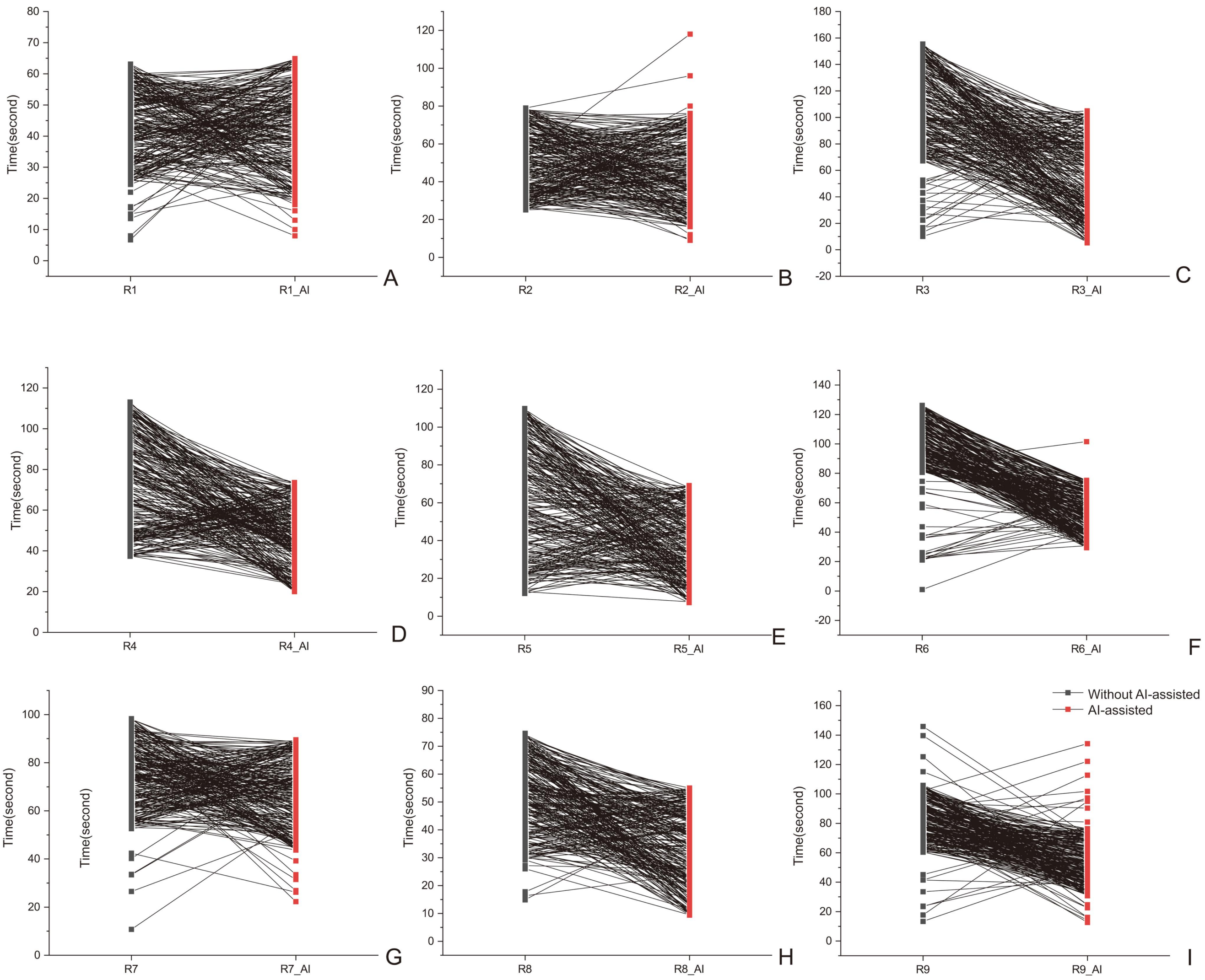

Supplementary Figure 10. Detailed architecture and workflow of the ProAI classification model

(A) HarDNet-39DS network architecture showing main layers and connections. (B) Input data processing pipeline demonstrating spatial standardization and ROI extraction. (C) Dual-source training data strategy integrating expert annotations and model-generated samples. (D) Case-level probability calculation method for single and multiple lesion scenarios.

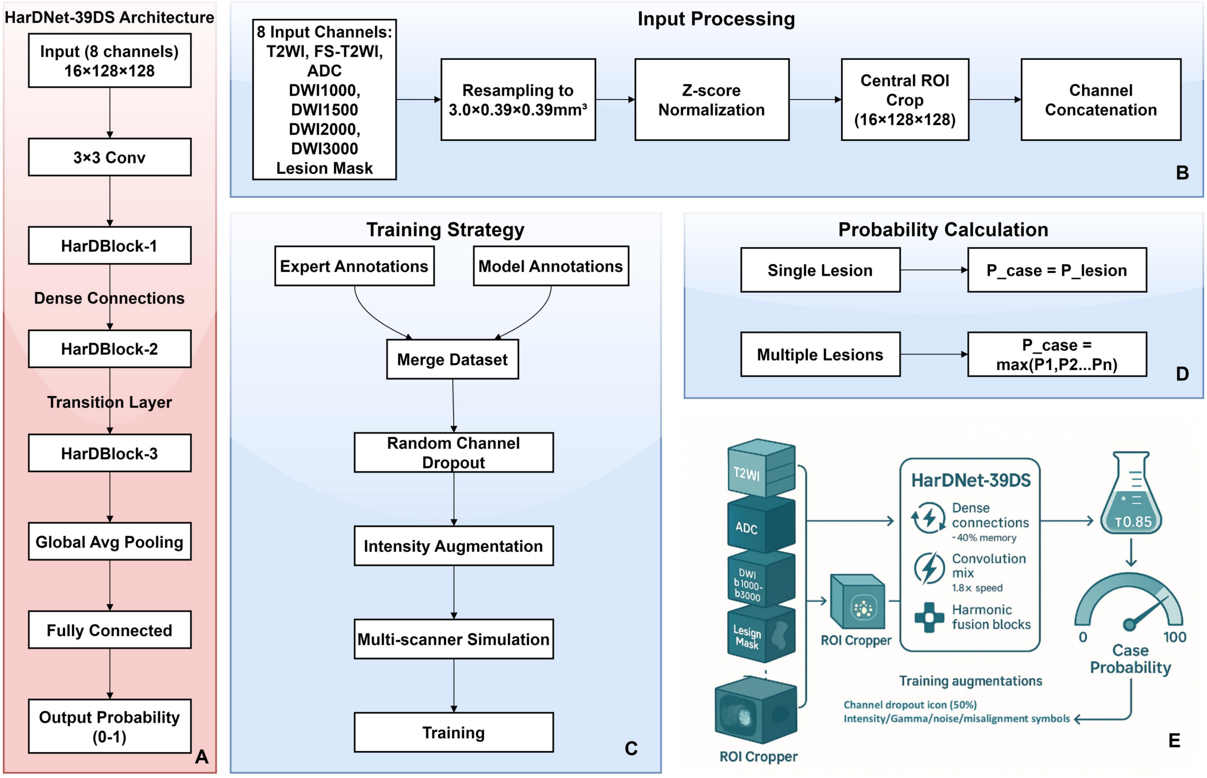

Supplementary Figure 11. Illustrates the system architecture and integration pathways, along with screenshots of the user interface in different integration scenarios.

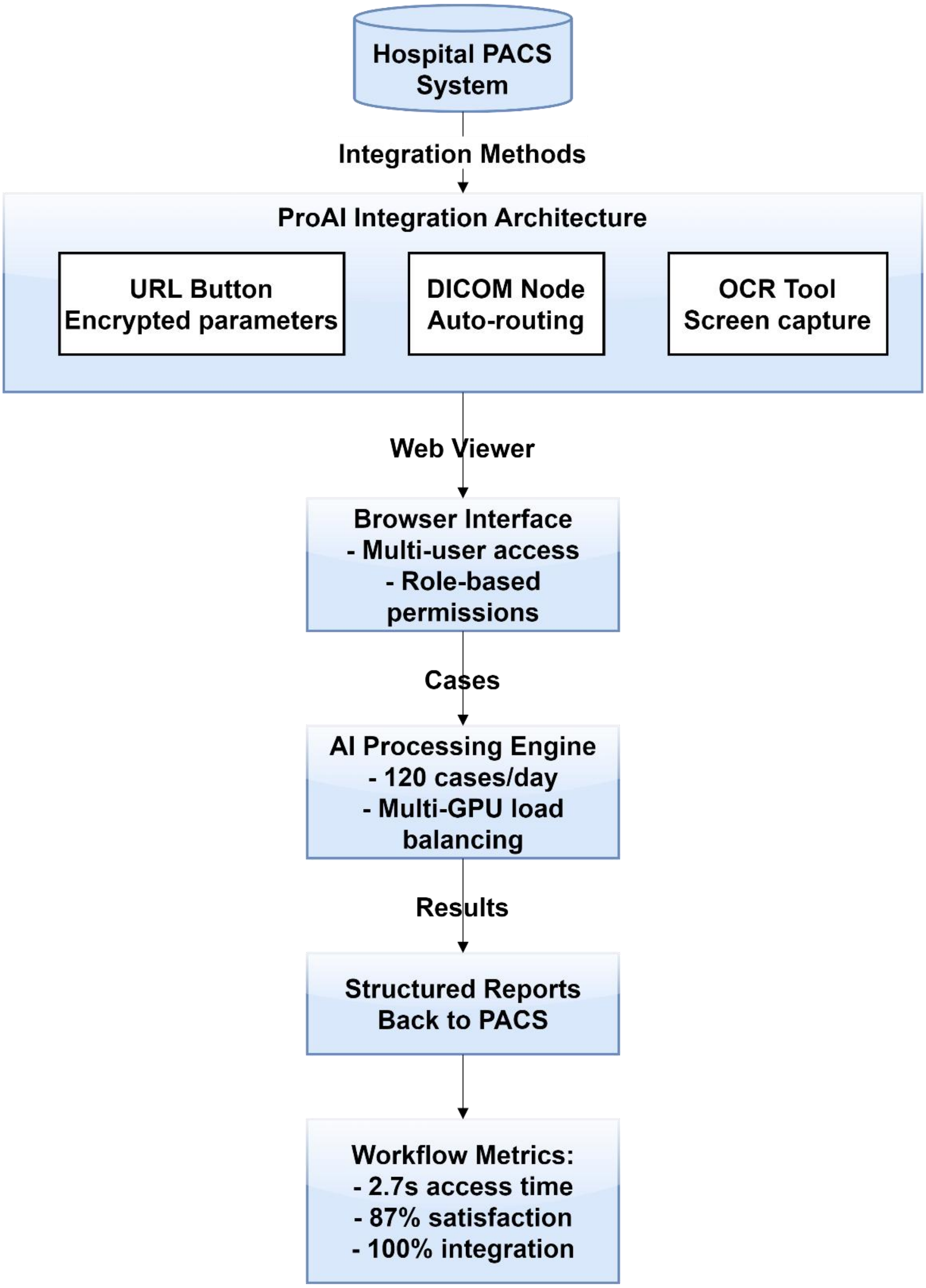

Supplementary Table 1. Comparison of Dice Values for Three Segmentation Algorithms

Supplementary Table 1. Comparison of Dice Values for Three Segmentation Algorithms

		nn-Unet		nn-Sam		LightM-UNet	
**Prostate**	Dataset	Median	Q1, Q3	Median	Q1, Q3	Median	Q1, Q3
	Training	0.956	0.949, 0.964	0.936	0.935, 0.937	0.894	0.793, 0.942
	Validation	0.931	0.926, 0.939	0.913	0.904, 0.921	0.891	0.856, 0.924
	All test	0.925	0.836, 0.951	0.885	0.847, 0.909	0.852	0.827, 0.868
	Test 1	0.957	0.943, 0.963	0.906	0.894, 0.918	0.86	0.847, 0.871
	Test 2	0.769	0.69, 0.867	0.818	0.762, 0.875	0.819	0.773, 0.862
	Test 3	0.794	0.674, 0.869	0.818	0.738, 0.858	0.794	0.726, 0.881
	Test 4	0.813	0.571, 0.902	0.77	0.692, 0.885	0.787	0.73, 0.832
	Test 5	0.92	0.874, 0.94	0.877	0.858, 0.899	0.853	0.836, 0.868
**Lesion**	Training	0.734	0.401, 0.867	0.647	0.473, 0.71	0.528	0.346, 0.556
	Validation	0.764	0.543, 0.889	0.551	0.486, 0.672	0.444	0.264, 0.571
	All test	0.564	0.388, 0.73	0.533	0.41, 0.626	0.504	0.317, 0.618
	Test 1	0.626	0.48, 0.774	0.538	0.477, 0.602	0.517	0.399, 0.614
	Test 2	0.479	0.389, 0.531	0.406	0.272, 0.562	0.344	0.214, 0.508
	Test 3	0.433	0.362, 0.535	0.451	0.327, 0.636	0.332	0.213, 0.484
	Test 4	0.709	0.48, 0.779	0.545	0.309, 0.742	0.392	0.201, 0.605
	Test 5	0.597	0.272, 0.758	0.579	0.481, 0.653	0.553	0.453, 0.663
**PI-RADS**	1-2	0	0, 0.23	0.375	0, 0.511	0.336	0, 0.473
	3	0.457	0.343, 0.557	0.472	0.295, 0.54	0.4	0.241, 0.51
	4	0.633	0.486, 0.734	0.567	0.5, 0.6	0.535	0.4, 0.586
	5	0.716	0.586, 0.863	0.65	0.544, 0.733	0.654	0.519, 0.746

Supplementary Table 2. Detection Rates by PI-RADS ≥ 3 and Lesion Characteristics in the test set 1-5 (Patient Level, n=515).

Supplementary Table 2: Detection Rates by PI-RADS Category and Lesion Characteristics in the test set 1-5 (Patient Level, n=515).

Category	Total Lesions	Detected	Detection Rate (%)
Detection by PI-RADS Category			
PI-RADS 3	173	152	87.9
PI-RADS 4	138	133	96.4
PI-RADS 5	204	200	98.0
All	515	485	94.2
Detection by Lesion Size			
<7mm	67	63	94.0
7-15mm	283	267	94.3
>15mm	165	155	93.9
Detection by Lesion Location			
Peripheral zone	330	322	97.6
Transition zone	144	135	93.8
Central zone	41	28	68.3

Supplementary Table 3. Segmentation Precision of Detected Lesions by PI-RADS ≥ 3 in the Test Set 1-5 (Patient Level, n=485).

Supplementary Table 3: Segmentation Precision of Detected Lesions by PI-RADS≥3 in the Test Set 1-5 (Patient Level, n=485).

Category	High Precision (DSC>0.7)	Moderate Precision (DSC 0.4-0.7)	Low Precision (DSC 0.1-0.4)
PI-RADS 3	31.6% (48/152)	37.5% (57/152)	30.9% (47/152)
PI-RADS 4	42.1% (56/133)	35.3% (47/133)	22.6% (30/133)
PI-RADS 5	49.0% (98/200)	33.5% (67/200)	17.5% (35/200)

Supplementary Table 4. Distribution and Diagnostic Efficacy of Lesions with Different Dice Values in Test Set 1-5 (Patient Level, n=611).

Supplementary Table 4 - Distribution and Diagnostic Efficacy of Lesions with Different Dice Values in Test Set 1-5 (Patient Level, n=611).

Dataset	N (csPca, %)	Model	AUC_CI	statistic	P value
DSC<0.3	139 (25, 18%)	ProAI	0.959 (0.923-0.996)	1.852	0.064
		PI-RADS	0.896 (0.828-0.964)		
DSC＜0.4	167 (37, 22%)	ProAI	0.921 (0.874-0.968)	1.432	0.152
		PI-RADS	0.874 (0.814-0.935)		
DSC＜0.5	259 (91, 35%)	ProAI	0.896 (0.856-0.937)	0.997	0.319
		PI-RADS	0.872 (0.828-0.915)		
DSC all	611(349, 57%)	ProAI	0.914 (0.892-0.937)	1.097	0.273
		PI-RADS	0.899 (0.874-0.924)		

Note, A total of 839 cases was included across Test Sets 1−5, of which 611 cases with radiology-delineated lesions were subjected to analysis.

All statistical tests were two-sided without multiplicity correction, with $p<0.05$ indicative of a statistically significant difference.

Supplementary Table 5. Comparison of lesion-level diagnostic performance between the AI model and PI-RADS in test datasets (Lesion Level, n=2,107)

Supplementary Table 5. Comparison of lesion-level diagnostic performance between the AI model and PI-RADS in test datasets (Lesion Level, n=2,107).

	AUC (95%CI)	Z value	P value[a]	Sensitivity	Specificity	Accuracy	PPV	NPV
**AI model**								
**Validation**	0.89(0.87-0.92)	-	-	0.89(256/288)	0.77(262/339)	0.83(518/627)	0.77(256/333)	0.89(262/294)
**Test set 1-5**	0.94(0.92-0.96)	-	-	0.91(336/368)	0.88(634/723)	0.89(970/1091)	0.79(336/425)	0.95(634/666)
**Test set 1**	0.93(0.90-0.96)	-	-	0.92(116/126)	0.83(151/181)	0.87(267/307)	0.80(116/146)	0.94(151/161)
**Test set 2**	0.89(0.84-0.94)	-	-	0.87(66/76)	0.81(117/144)	0.83(183/220)	0.71(66/93)	0.92(117/127)
**Test set 3**	0.92(0.83-1.00)	-	-	0.83(10/12)	0.91(38/42)	0.89(48/54)	0.71(10/14)	0.95(38/40)
**Test set 4**	0.93(0.88-0.99)	-	-	0.91(19/21)	0.82(42/51)	0.85(61/72)	0.68(19/28)	0.96(42/44)
**Test set 5**	0.97(0.95-0.99)	-	-	0.94(125/133)	0.94(286/305)	0.94(411/438)	0.87(125/144)	0.97(286/294)
**Test set TCIA**	0.86(0.82-0.90)			0.87(137/158)	0.69(104/151)	0.78(241/309)	0.75(137/184)	0.83(104/125)
PI-RADS								
**Validation**	0.93(0.91-0.95)	3.265	0.001	0.97(287/295)	0.52(132/256)	0.76(419/551)	0.70(287/411)	0.94(132/140)
**Test set 1-5**	0.93(0.91-0.95)	0.405	0.685	0.98(364/373)	0.60(303/501)	0.76(667/874)	0.65(364/562)	0.97(303/312)
**Test set 1**	0.93(0.90-0.96)	0.64	0.522	0.98(126/128)	0.59(79/135)	0.78(205/263)	0.69(126/182)	0.98(79/81)
**Test set 2**	0.84(0.79-0.90)	0.67	0.503	0.97(76/78)	0.40(33/83)	0.68(109/161)	0.60(76/126)	0.94(33/35)
**Test set 3**	0.93(0.87-1.00)	0.513	0.608	1.00(12/12)	0.62(18/29)	0.73(30/41)	0.52(12/23)	1.00(18/18)
**Test set 4**	0.92(0.85-0.99)	0.311	0.756	1.00(21/21)	0.46(16/35)	0.66(37/56)	0.52(21/40)	1.00(16/16)
**Test set 5**	0.96(0.94-0.98)	0.625	0.532	0.96(129/134)	0.72(157/219)	0.81(286/353)	0.68(129/191)	0.97(157/162)
**Test set TCIA**	0.82(0.77-0.87)	1.057	0.291	0.94(152/162)	0.32(34/105)	0.70(186/267)	0.68(152/223)	0.77(34/44)

AUC = area under the curve; CI = confidence interval; PI-RADS = prostate imaging reporting and data system; PPV = positive predictive value; NPV = negative predictive value

[a] P values calculated from the DeLong test between AI model and PI-RADS scores. All statistical tests were two-sided, with p<0.05 indicative of a statistically significant difference.

Note: AUCs are calculated from continuous scores. For the AI model, sensitivity/specificity/accuracy/PPV/NPV in each test set are computed at a per-dataset threshold selected by maximizing Youden's J; for PI-RADS, a fixed threshold of ⩾3 is used. The pooled 'Test set 1-5' metrics are micro-averaged by summing TP/FP/TN/FN across the datasets at their respective thresholds.

Supplementary Table 6. Subgroup Analysis of ProAI and PI-RADS Scores by T2WI/DWI Spatial Resolution, Grouping Criteria, and Machine Model in the Test Set 1-5 and TCIA (Patient Level, n=879).

Supplementary Table 6. Subgroup Analysis of ProAI and PI-RADS Scores by T2WI/DWI Spatial Resolution, Grouping Criteria, and Machine Model in the Test Set 1-5 and TCIA (Patient Level, n=879).

	In-Plane Resolution (mm^2)	Manufacturer	Model Name
**T2WI**			
High-resolution (n=325)	0.39 - 0.449	GE (n=249), Philips (n=40), SIEMENS (n=19), UIH (n=17)	DISCOVERY MR750w, Signa HDxt, SIGNA Architect, Optima MR450w; Ingenia; Skyra, Avanto_fit; uMR 790;
Median-resolution (n=266)	0.45 - 0.59	GE (n=229), Philips (n=36), UIH (n=1)	DISCOVERY MR750, DISCOVERY MR750w, Signa HDxt, SIGNA; Ingenia CX; uMR 790
Low-resolution (n=288)	> 0.60	SIEMENS (n=282), GE (n=4), Philips (n=2)	MAGNETOM Vida, Prisma, Prisma_fit, Skyra, Skyra_fit, TrioTim; Signa HDxt, DISCOVERY MR750; Achieva;
**DWI**			
High-resolution (n=202)	0.39 - 1.39	GE (n=176), UIH (n=18), SIEMENS (n=2), Philips (n=6)	Signa HDxt, DISCOVERY MR750, DISCOVERY MR750w, Optima MR450w, SIGNA Architect; uMR 790; Prisma; Ingenia CX
Median-resolution (n=337)	1.40 – 1.59	GE (n=293), Philips (n=30), SIEMENS (n=14)	DISCOVERY MR750w, DISCOVERY MR750, Signa HDxt, SIGNA; Ingenia CX; Skyra
Low-resolution (n=340)	> 1.60	SIEMENS (n=285), Philips (n=42), GE (n=13)	Skyra, MAGNETOM Vida, Prisma, Skyra_fit, Prisma_fit, TrioTim; Ingenia, Ingenia CX, Achieva; Signa HDxt, DISCOVERY MR750

Supplementary Table 7. T2WI Resolution-Based Performance Analysis in the Test Set 1-5 and TCIA (Patient Level, n=879).

Supplementary Table 7: T2WI Resolution-Based Performance Analysis in the Test Set 1-5 and TCIA (Patient Level, n=879).

Resolution (mm²)	Group	N	AUC (95% CI)	P value	Sensitivity	P value	Specificity	P value	PPV	P value	NPV	P value	Accuracy	P value
High (≤0.45)	ProAI	325	0.92 (0.89,0.95)		0.84		0.88		0.88		0.85		0.86	
	PI-RADS	325	0.91 (0.88,0.94)		0.8		0.9		0.89		0.82		0.85	
Medium (0.45-0.59)	ProAI	266	0.88 (0.83,0.92)	0.087	0.87	0.51	0.77*	0.027	0.9	0.538	0.71*	0.01	0.84	0.431
	PI-RADS	266	0.93 (0.9,0.97)	0.301	0.88*	0.046	0.89	0.729	0.95	0.059	0.75	0.232	0.88	0.284
Low (≥0.6)	ProAI	288	0.78 (0.72,0.83) *	<0.001	0.75*	0.037	0.68*	<0.001	0.73*	0.001	0.7*	0.003	0.71*	<0.001
	PI-RADS	288	0.85 (0.81,0.89) *	0.032	0.81	0.729	0.83	0.061	0.85	0.256	0.79	0.583	0.82	0.321

Note: ProAI = the clinically significant prostate cancer artificial intelligence model; AUC = area under the curve; CI = confidence interval; PI-RADS = prostate imaging reporting and data system; PPV = positive predictive value; NPV = negative predictive value

P value was compared to high-resolution group, * represent p<0.05. All statistical tests were two-sided without multiplicity correction, with p<0.05 indicative of a statistically significant difference.

Supplementary Table 8. DWI Resolution Based Performance Analysis in the Test Set 1-5 and TCIA (Patient Level, n=879).

Supplementary Table 8: DWI Resolution Based Performance Analysis in the Test Set 1-5 and TCIA (Patient Level, n=879).

Parameter	Group	N	AUC (95% CI)	P value	Sensitivity	P value	Specificity	P value	PPV	P value	NPV	Accuracy	P value
High Resolution (≤1.38 mm^2)	ProAI	202	0.89 (0.85,0.94)		0.91		0.77		0.89		0.79	0.86	
	PI-RADS	202	0.92 (0.87,0.96)		0.90		0.86		0.93		0.80	0.89	
Medium Resolution (1.4 - 1.59 mm^2)	ProAI	337	0.92 (0.89,0.95)	0.424	0.84	0.026	0.88	0.957	0.90	0.544	0.83	0.86	0.978
	PI-RADS	337	0.92 (0.9,0.95)	0.802	0.79*	0.269	0.91	0.51	0.91	0.871	0.79	0.85	0.189
Low Resolution (≥1.6 mm^2)	ProAI	340	0.80 (0.75,0.84) *	0.005	0.76*	0.296	0.70	0.001	0.75*	0.227	0.71	0.73*	<0.001
	PI-RADS	340	0.86 (0.82,0.9)	0.074	0.82	0.674	0.84	0.044	0.86*	0.972	0.80	0.83	0.073

Note: AI = artificial intelligence; AUC = area under the curve; CI = confidence interval; PI-RADS = prostate imaging reporting and data system; PPV = positive predictive value; NPV = negative predictive value

P value was compared to high-resolution group, * represent p<0.05. All statistical tests were two-sided without multiplicity correction, with p<0.05 indicative of a statistically significant difference.

Supplementary Table 9. Field Strength Performance Analysis in the Test Set 1-5 (Patient Level, n=839).

Supplementary Table 9: Field Strength Performance Analysis in the Test Set 1-5 (Patient Level, n=839).

Parameter	Group	N	AUC (95% CI)	Sensitivity	Specificity	PPV	NPV	Accuracy
1.5T	ProAI	179	0.89 (0.84-0.93)	0.85	0.82	0.80	0.87	0.83
	PI-RADS	179	0.88 (0.83-0.92)	0.83	0.84	0.81	0.86	0.84
3.0T	ProAI	660	0.92 (0.90-0.94)	0.90	0.85	0.83	0.91	0.87
	PI-RADS	660	0.92 (0.90-0.94)	0.87	0.88	0.85	0.89	0.87

Note: AI = artificial intelligence; AUC = area under the curve; CI = confidence interval; PI-RADS = prostate imaging reporting and data system; PPV = positive predictive value; NPV = negative predictive value $p>0.05$ for all comparisons between field strength groups and between coil configuration groups

Supplementary Table 10. Diagnostic performance in the MRMC study (Patient Level, n=250).

Supplementary Table 10. Diagnostic performance in the MRMC study (Patient Level, n=250).

Readers		AUC	95%CI		AUC improvement	Z	P value
R1	Without AI	0.87	0.82	0.91	<0.01	0.15	0.89[a]
	With AI	0.87	0.82	0.92			
R2	Without AI	0.90	0.86	0.94	0.02	1.39	0.19[a]
	With AI	0.92	0.89	0.96			
R3	Without AI	0.82	0.77	0.88	0.05	1.92	0.06[a]
	With AI	0.87	0.83	0.92			
R4	Without AI	0.79	0.74	0.85	0.06	2.63	＜0.01[a]
	With AI	0.86	0.80	0.91			
R5	Without AI	0.64	0.57	0.71	0.16	4.16	＜0.01[a]
	With AI	0.81	0.75	0.86			
R6	Without AI	0.82	0.77	0.88	0.06	2.32	0.02[a]
	With AI	0.88	0.84	0.93			
R7	Without AI	0.78	0.73	0.84	0.06	2.52	0.01[a]
	With AI	0.84	0.79	0.89			
R8	Without AI	0.81	0.75	0.86	0.06	2.13	0.03[a]
	With AI	0.87	0.82	0.91			
R9	Without AI	0.78	0.72	0.84	0.09	3.62	＜0.01[a]
	With AI	0.87	0.83	0.92			
Senior radiologists	Without AI	0.88	0.82	0.95	0.01	1.28	0.42[b]
	With AI	0.89	0.74	1.0			
Urologists	Without AI	0.82	0.78	0.87	0.05	3.01	＜0.01[b]
	With AI	0.88	0.84	0.92			
General radiologists	Without AI	0.76	0.68	0.84	0.09	4.15	＜0.01[b]
	With AI	0.85	0.80	0.89			
Average	Without AI	0.80	0.74	0.86	0.06	3.87	＜0.01[b]
	With AI	0.86	0.82	0.90			

AI = artificial intelligence; AUC = area under the curve; CI = confidence interval; PI-RADS = prostate imaging reporting and data system

[a] P value was calculated by DeLong test.

[b] P value was calculated by OR method.

All statistical tests were two-sided without multiplicity correction, with $p<0.05$ indicative of a statistically significant difference.

Supplementary Table 11. Diagnostic Performance of PI-RADS in MRMC study (Patient Level, n=250).

Supplementary Table 11. Diagnostic Performance of PI-RADS in MRMC study (Patient Level, n=250).

	AUC (95%CI)	Sensitivity	Specificity	Accuracy	PPV	NPV
R1 without AI	0.87 (0.82-0.91)	0.87(102/117)	0.74(99/133)	0.80(201/250)	0.75(102/136)	0.87(99/114)
R1 with AI	0.87 (0.83-0.91)	0.91(106/117)	0.71(94/133)	0.80(200/250)	0.73(106/145)	0.90(94/105)
R2 without AI	0.90 (0.86-0.94)	0.91(106/117)	0.75(100/133)	0.82(206/250)	0.76(106/139)	0.90(100/111)
R2 with AI	0.92 (0.89-0.96)	0.95(111/117)	0.67(89/133)	0.80(200/250)	0.72(111/155)	0.94(89/95)
R3 without AI	0.82 (0.77-0.88)	0.81(95/117)	0.70(93/133)	0.75(188/250)	0.70(95/135)	0.81(93/115)
R3 with AI	0.87 (0.83-0.91)	0.91(106/117)	0.71(95/133)	0.80(201/250)	0.74(106/144)	0.90(95/106)
R4 without AI	0.79 (0.74-0.85)	0.91(107/117)	0.41(54/133)	0.64(161/250)	0.58(107/186)	0.84(54/64)
R4 with AI	0.86 (0.81-0.90)	0.86(101/117)	0.69(92/133)	0.77(193/250)	0.71(101/142)	0.85(92/108)
R5 without AI	0.64 (0.58-0.71)	0.72(84/117)	0.50(67/133)	0.60(151/250)	0.56(84/150)	0.67(67/100)
R5 with AI	0.81 (0.75-0.86)	0.84(98/117)	0.64(85/133)	0.73(183/250)	0.67(98/146)	0.82(85/104)
R6 without AI	0.82 (0.77-0.88)	0.84(98/117)	0.73(97/133)	0.78(195/250)	0.73(98/134)	0.84(97/116)
R6 with AI	0.88 (0.84-0.93)	0.92(108/117)	0.74(99/133)	0.83(207/250)	0.76(108/142)	0.92(99/108)
R7 without AI	0.78 (0.73-0.84)	0.88(103/117)	0.40(53/133)	0.62(156/250)	0.56(103/183)	0.79(53/67)
R7 with AI	0.84 (0.80-0.89)	0.88(103/117)	0.63(84/133)	0.75(187/250)	0.68(103/152)	0.86(84/98)
R8 without AI	0.81 (0.76-0.86)	0.82(96/117)	0.61(81/133)	0.71(177/250)	0.65(96/148)	0.79(81/102)
R8 with AI	0.87 (0.82-0.91)	0.95(111/117)	0.67(89/133)	0.80(200/250)	0.72(111/155)	0.94(89/95)
R9 without AI	0.78 (0.72-0.83)	0.85(100/117)	0.56(75/133)	0.70(175/250)	0.63(100/158)	0.82(75/92)
R9 with AI	0.87 (0.83-0.91)	0.94(110/117)	0.57(76/133)	0.74(186/250)	0.66(110/167)	0.92(76/83)
ProAI alone	0.90 (0.86-0.94)	0.88(103/117)	0.78(104/133)	0.83(207/250)	0.78(103/132)	0.88(104/118)

AI = artificial intelligence; AUC = area under the curve; CI = confidence interval; PI-RADS = prostate imaging reporting and data system; PPV = positive predictive value; NPV = negative predictive value.

Supplementary Table 12. The expertise of individual readers in the MRMC study (Patient Level, n=250).

Supplementary Table 12. The expertise of individual readers in the MRMC study (Patient Level, n=250).

Reader ID	Experience(year)	MR diagnosis per year	Prostate MR diagnosis per year	Qualification category
Reader 1 (R 1)	19	6000	1200	Senior radiologist
Reader 2 (R 2)	15	5500	1100	Senior radiologist
Reader 3 (R 3)	16	-	1200[a]	Urologist
Reader 4 (R 4)	15	10000	500	General radiologist
Reader 5 (R 5)	9	2000	50	General radiologist
Reader 6 (R 6)	6	-	500[a]	Urologist
Reader 7 (R 7)	4	1200	60	General radiologist
Reader 8 (R 8)	3	4800	200	General radiologist
Reader 9 (R 9)	4	2400	110	General radiologist

Specialists were proficient in abdominal radiology, with over 5 years of experience in prostate diagnosis, having evaluated more than 3000 cases. General radiologists across diverse experience levels and no specialization in prostate diagnosis. Urologists are frontline physicians in performing MRI-guided prostate biopsy with more than 5 years experiences.

[a] Urologists' prostate MRI diagnoses correlate with the number of prostate biopsies performed.

Supplementary Table 13. Technology Acceptance Model (TAM) Questionnaire Results

Supplementary Table 13. Technology Acceptance Model (TAM) Questionnaire Results

Item	Mean Score (1-5)	SD
Perceived Usefulness		
1. Using ProAI improves my diagnostic accuracy	4.4	0.7
2. Using ProAI enhances my diagnostic efficiency	4.5	0.6
3. ProAI makes it easier to identify csPCa	4.3	0.8
4. I find ProAI useful in my diagnostic workflow	4.2	0.7
Perceived Ease of Use		
5. Learning to operate ProAI is easy	4.1	0.8
6. Integration of ProAI into my workflow is clear	3.8	0.9
7. Using ProAI requires minimal mental effort	3.7	1.0
8. I find ProAI easy to use	4.0	0.7
Intention to Use		
9. I would use ProAI regularly in clinical practice	4.6	0.6
10. I would recommend ProAI to colleagues	4.4	0.7

Supplementary Table 14. Analysis of Diagnostic Inconsistencies Between ProAI and Radiologists in High-Risk Prospective Cohort (Patient Level, n=1,051).

Supplementary Table 14. Analysis of Diagnostic Inconsistencies Between ProAI and Radiologists in High-Risk Prospective Cohort (Patient Level, n=1,051).

Category	Number of Cases	Percentage	Description	Biopsy Performed	Confirmed csPCa
Subtle DWI Findings	9	45.0%	Small lesions (<1 cm) with mild diffusion restriction below AI detection threshold	5	2
Complex Anatomic Regions	6	30.0%	Challenging locations (prostatic base: 4, anterior transition zone: 2)	3	1
Technical Factors	3	15.0%	Image artifacts (DWI susceptibility: 2, motion: 1)	2	0
Atypical Presentation	2	10.0%	Heterogeneous signal or unusual morphology	1	0
Total	20	100%		11 (55%)	3 (27.3%)

Supplementary Table 15. Analysis of Diagnostic Inconsistencies Between ProAI and Radiologists in Low-Risk Prospective Cohort (Patient Level, n=1,051).

Supplementary Table 15. Analysis of Diagnostic Inconsistencies Between ProAI and Radiologists in Low-Risk Prospective Cohort (Patient Level, n=1,051).

Category	Number of Cases	Percentage	Description	Biopsy Performed	Confirmed csPCa
Transitional Zone Lesions	22	39.3%	Difficult distinction between BPH nodules and cancer	18	8
Borderline PI-RADS 3 vs 4	19	33.9%	Features at boundary between PI-RADS categories	14	5
Prostatitis Mimics	8	14.3%	Inflammatory changes simulating malignancy	5	2
Hemorrhagic Changes	5	8.9%	Post-biopsy or other hemorrhage	3	1
Technical Limitations	2	3.6%	Suboptimal image quality	2	1
Total	56	100%		42 (75%)	17 (40.5%)

Supplementary Table 16. Scanning machines and scanning parameters of all datasets (Patient Level, n=7,849).

Supplementary Table 16. Scanning machines and scanning parameters of all datasets (Patient Level, n=7,849).

**Parameter**	**Training Set**				**Validation Set**	**Test Set. 1**	**Test Set. 2**	**Test Set. 3**	**Test Set. 4**	**Test Set. 5**	**Test Set. TCIA**	**Real-world Set (before ProAI)**	**Real-world Set (after ProAI)**
	Hospital 1	PI-CAI study			Hospital 1	Hospital 2	Hospital 3	Hospital 4	Hospital 5	Hospital 6	TCIA	Hospital 1	Hospital 1
**No. of cases**	2,794	800	350	350	478	246	156	41	51	345	260	927	1,051
**MRI scanners**	3	4	1	6	2	1	4	3	1	2	10	2	2
**Manufacturer**	GE, SIEMENS	SIEMENS	SIEMENS	SIEMENS, Philips	GE	GE	Philips, GE, SIEMENS	Philips, GE, UIH	Philips	GE, SIEMENS	Philips, GE, SIEMENS	GE	GE
**Model**	MR750, SIGNA Premier, Skyra	Avanto, Prisma, Skyra, TrioTim	Skyra	Achieva, Aera, Avanto, Ingenia, Prsma, Skyra	MR750, SIGNA Premier	MR750w	Ingenia CX, Signa HDxt, Prisma	Ingenia CX, Signa, uMR 790	Ingenia	MR750, Skyra	Achieva, Avanto fit, MR750w, MAGNETOM Vida, Optima MR450w, Prisma fit, SIGNA Architect, Skyra, Skyra fit, TrioTim	MR750, SIGNA Premier	MR750, SIGNA Premier
**Field Strengths (Tesla)**	3	1.5, 3	3	1.5, 3	3	3	3	3	3	3	1.5, 3	3	3
**T2W Acquisition**													
**In-Plane Resolution (mm)**	0.39-0.78	0.3-0.6	0.3	0.2-0.8	0.39	0.43	0.54-0.625	0.39-0.57	0.31	0.375-0.59	0.33-0.66	0.39	0.39
**Slice Thickness (mm)**	3-4	3-5	3.6	2.2-3.6	3-4	4	3-4	3.5-4	3	3-4.5	1.5-3	3-4	3-4
**DWI/ADC Acquisition**													
**In-Plane Resolution (mm^2)**	1.25-1.86	2-2.5	2	0.9-1.9	1.25	1.4	1.0-1.7	0.39-1.4	1.7	1.2-1.6	0.86-2.34	1.25	1.25

**Slice Thickness (mm)**	3-5	3-4.5	3.6	3-5	3-5	4	3-4	3-4	3	3-4.5	3-4	3-5	3-5
**High b-Value (s/mm²)**	1000, 1500,2000, 3000	1000, 1400	1400	1400, 1500, 2000	1000, 1500,20 00,3000	1000	1000, 1400, 2000	1000, 1500, 2000, 2500	1,500	1000, 1200, 1500	1000,1400	1000, 1500,2000,300 0	1000, 1500,2000,300 0
**DCE Acquisition**													
**In-Plane Resolution (mm²)**	0.7-1.3	NA	NA	NA	0.7-1.3	1	1.3-1.4	0.9-1.1	1	1-1.2	NA	0.7-1.3	0.7-1.3
**Slice Thickness (mm)**	1-1.5	NA	NA	NA	1	1.2	3	1.5-1.7	1	1, 1.5	NA	1	1
**temporal resolution(second)**	10,15, 20	NA	NA	NA	10,15	10	10	11, 6, 28	16	18, 24	NA	10,15	10,15

ADC = Apparent diffusion coefficient; DCE = Dynamic contrast enhancement; DWI = Diffusion-weighted imaging; MRI = Magnetic resonance imaging; PPA = pelvic phased-array surface coil; T2W = T2-weighted imaging;
