# Supplementary material for: Automated MRI system for clinically significant prostate cancer detection development validation and real-world implementation
Source: Nat Commun. 2025 Nov 23;16:11583. doi: 10.1038/s41467-025-66593-z (PMC12749140; doi:10.1038/s41467-025-66593-z)
Supplement: Supplementary file 2 — Description of Additional Supplementary Files [file 41467_2025_66593_MOESM2_ESM.pdf]

Description of Additional Supplementary Files

File Name: Supplementary Movie 1

Description: Detailed operating procedures of ProAI, including image upload steps, total processing time, and software interface descriptions.
